# Inferring the spread of COVID-19: the role of time-varying reporting rate in epidemiological modelling

**DOI:** 10.1038/s41598-022-14979-0

**Published:** 2022-06-24

**Authors:** Adam Spannaus, Theodore Papamarkou, Samantha Erwin, J. Blair Christian

**Affiliations:** 1 Computational Sciences and Engineering Division, Oak Ridge National Laboratory, Oak Ridge, TN USA; 2 Department of Mathematics, The University of Manchester, Manchester, UK; 3 Department of Mathematics, University of Tennessee, Knoxville, TN USA; 4 Pacific Northwest National Laboratory, Richland, WA USA

**Keywords:** Applied mathematics, Computational models

## Abstract

The role of epidemiological models is crucial for informing public health officials during a public health emergency, such as the COVID-19 pandemic. However, traditional epidemiological models fail to capture the time-varying effects of mitigation strategies and do not account for under-reporting of active cases, thus introducing bias in the estimation of model parameters. To infer more accurate parameter estimates and to reduce the uncertainty of these estimates, we extend the SIR and SEIR epidemiological models with two time-varying parameters that capture the transmission rate and the rate at which active cases are reported to health officials. Using two real data sets of COVID-19 cases, we perform Bayesian inference via our SIR and SEIR models with time-varying transmission and reporting rates and via their standard counterparts with constant rates; our approach provides parameter estimates with more realistic interpretation, and 1-week ahead predictions with reduced uncertainty. Furthermore, we find consistent under-reporting in the number of active cases in the data that we consider, suggesting that the initial phase of the pandemic was more widespread than previously reported.

## Introduction

During a disease outbreak, epidemiological forecasting informs public health decisions by predicting how widely the disease will possibly spread^1^. Such information is critical for public health officials trying to understand the dynamics through which a disease is transmitted and to slow its propagation^2^. It is also important to quantify the proportion of infections in a community and the frequency with which infections are being reported to public health officials. Moreover, in the case of a novel disease, there are uncertainties in estimates of the infection rate and of the incubation period, and reliable testing strategies have yet to be developed. These considerations are relevant during the SARS-CoV-2 (COVID-19) pandemic as the uncertainty surrounding the disease’s transmission and incubation rates have made coordinating the global response to this disease a substantial challenge to public health officials.

One of the most widely employed mathematical models of general disease transmission is the Susceptible-Infected-Removed (SIR) model^3^. This model describes the interactions between three population groups: those who are are susceptible to an infection, those who are infected, and those who have been removed from the population through either recovery or death. Over time, the SIR model has been augmented to include other groups, such as an exposed individuals, asymptomatic carriers, or individuals immune to the infection^4^. In the present context of the COVID-19 pandemic, a latency period in the course of the disease has been proposed^5^, during which an individual carrying the disease does not present symptoms. This suggests the possibility of including an ‘exposed’ state, thus modelling the spread of COVID-19 with a Susceptible-Exposed-Infected-Resistant (SEIR) model.

Deterministic epidemiological models cannot reflect the real-time implications of preventative measures, such as safer-at-home orders, social distancing, or mask mandates that directly impact the transmission rate^6^. Moreover, these models do not take into account climatic changes, nor social cycles, e.g., holiday gatherings or the periodic starting and stopping of the school calendar. To capture the time-varying effects of preventative measures and changes in social interactions, we choose stochastic state space representations of both the SIR and SEIR model and adopt a Bayesian approach.

The novelty in our model is that the rate of transmission and the reporting rate of positive cases vary in time. Time-varying transmission and reporting rates, as opposed to constant ones, provide more flexible modelling assumptions, and thus adhere more closely to respective observed rates in COVID-19 data. By allowing the transmission rate to vary in time, our model adapts to heterogeneity of disease outcomes, to mutations in the disease, to asymptomatic transmission, and to mitigation strategies used to slow the spread of the disease. However, a time-varying transmission rate does not fully capture the spread of a disease though a population. Indeed, not all cases are reported due to faulty tests, inability to obtain a test, or false negative results for example. In order to further quantify disease proliferation through a susceptible population, we also allow the reporting rate to vary in time. By including a time-varying reporting rate, our model is more flexible to adapt to under-reporting of cases and to advancements in testing reliability. In other words, this time-varying reporting rate allows our model to dynamically adapt to changes in the spread of COVID-19, thus informing more accurately the reporting of positive cases to the health agencies.

During the COVID-19 pandemic, and specifically in the early phases, a lag in testing times and under-reported case rates have been observed. Similar studies on the Zika virus note a high occurrence of under-reporting and estimate reporting rates by including a separate unreported infected population in epidemiological modelling^7^. Similarly, in estimating the reporting rate for shigellosis, a secondary unreported infected compartment is proposed by Joh et al.^8^. An alternative approach is taken in a study of an influenza pandemic, where a functional form of the reporting rate is estimated as either linearly increasing or constant depending on the time-frame of the pandemic^9,10^. Rather than introducing an additional compartment into an epidemiological model, we instead employ a time-varying parameter of reporting rate. So, we avoid a structural change to our model, while making the model more flexible via time-varying parameterization. Augmenting an epidemiological model is not the only approach to estimate the spread of a disease throughout a population. Alternatively, one could develop an artificial neural network^11^, or a fractional-order epidemiological model^12^.

To demonstrate our approach, we study two regional outbreaks with different mitigation strategies using the COVID-19 case counts, as collated by the New York Times^13^. Considering data from Tennessee and New York showcases the flexibility of our Bayesian model with time-varying transmission and reporting rates, because the spread of COVID-19 in these states presents different modelling challenges. Notably, these two states experienced the initial 6 months of the pandemic in widely differing fashion. Indeed, each state handled the beginning of the pandemic differently, employed different testing strategies, and continue to have different reopening policies^14^. Such widely varying pandemic management strategies are demonstrated by the COVID-19 data sets associated with Tennessee and New York, which exhibit drastically different dynamics of case counts. Our experiments highlight the capacity of our Bayesian state space epidemiological models with time-varying transmission and reporting rates to fit data representing different dynamics of disease spread, to estimate the transmission rate and the reporting rate during the progression of COVID-19, and to reduce the uncertainty in predictions of COVID-19 cases.

## Model formulation

### S(E)IR epidemiological model

To investigate the spread of COVID-19 through a population, we use the traditional SIR and SEIR epidemiological models, augmenting them with two temporal variables. One of these variables accounts for the rate of transmission, while the other variable quantifies the rate at which positive cases are reported. These time-varying variables account for variation in the transmissibility of a disease and in the rate at which new infections are reported. Consequently, our model is able to account for variation and uncertainty present during a public health emergency.

Allowing the transmission rate of COVID-19 to vary in time, similar to the approach of^15–18^, we are able to capture intervention measures enacted by public health officials, such as mask mandates or shelter-in-place orders, or ‘super-spreader’ events which have direct impacts on disease prevalence. By modelling the rate at which the disease spreads as a time-varying variable, we may better quantify the spread of the disease, which in turn, yields more accurate information about the course of the pandemic to public health officials concerned with slowing community spread.

Quantifying the transmissibility of a disease does not fully capture its reach and spread, though. If we consider the early phases of the COVID-19 pandemic, wide-spread access to testing was unavailable, and disease prevalence within communities was widely under-reported^19,20^. To this end, we present a novel S(E)IR epidemiological model by introducing two time-varying variables, namely the *reporting rate* *p*(*t*) and the *transmission rate* $\beta (t)$. The reporting rate *p*(*t*) captures the percentage of positive cases reported, and the transmission rate $\beta (t)$ quantifies the rate at which COVID-19 spreads through a population at time *t*.

The SIR epidemiological model with time-varying transmission rate $\beta (t)$ is represented by the system of differential equations

1 $$\begin{aligned} \begin{aligned} {\mathrm{d}}S(t)&= -\beta (t)S(t)\frac{I(t)}{N} {\mathrm{d}}t, \\ {\mathrm{d}}I(t)&= (\beta (t)S(t)\frac{I(t)}{N} - \gamma I(t)) {\mathrm{d}}t,\\ {\mathrm{d}}R(t)&= \gamma I(t)\, {\mathrm{d}}t,\\ {\mathrm{d}}w(t)&= \mu (w(t), \theta _w) {\mathrm{d}}t + \sigma (w(t),\theta _w)\,{\mathrm{d}}B(t), \quad w(t) = g(\beta (t)), \end{aligned} \end{aligned}$$

where *w*(*t*) is a stochastic differential equation (SDE), which entails the Wiener process *B*(*t*) and controls the transmission rate^21^. In this SDE, we define drift $\mu$ and diffusion $\sigma$ terms, parameterized by $\theta _w$, and a function $g:{\mathbb {R}}^+\rightarrow {\mathbb {R}}$. It is this time-varying parameter that controls the extent to which a disease spreads throughout a population. Lastly, to fully specify the epidemiological model, we write *S*(*t*) as individuals susceptible to COVID-19, *I*(*t*) as individuals infected with COVID-19, and *R*(*t*) as individuals removed from the population who are no longer able to become infected. Susceptible individuals move into the infected compartment at rate $\beta (t)$ and infected individuals become removed at rate $\gamma$.

An expansion on the SIR model is the SEIR model which includes an additional ‘exposed’ compartment *E*(*t*) between the ‘susceptible’ and ‘infected’ compartments. Individuals who are in the disease’s latent period, the ‘exposed’ compartment, move into the ‘infected’ compartment with rate $\kappa$ and are and thus are capable of infecting other susceptible individuals. The system of differential equations that represents the SEIR model can be found in appendix A. The salient difference between the SIR and SEIR models is the inclusion of the ‘exposed’ state in the latter; individuals move into this compartment once they have been exposed to an infected individual, but are not yet expressing any symptoms. Such a latent phase is pertinent when quantifying the breadth of the pandemic due to the challenges in correctly accounting for such individuals. Moreover, the ‘exposed’ compartment not only acts as a delay between the ‘susceptible’ and ‘infected’ compartments, but individuals in this compartment may transmit the disease before becoming actively infected themselves.

In this study, for both SIR and SEIR models, we take $\mu =0$ and $\sigma (w(t),\theta _w)=\theta _w$, thus assuming that the transmission rate on any day is likely to be the same as the previous day, and set $g(\cdot ) = \log (\cdot )$. By choosing $\mu =0$ and $\sigma (w(t),\theta _w)=\theta _w$, the resulting path *w*(*t*), defined by the SDE ${\mathrm{d}}w(t)=\theta _w{\mathrm{d}}B(t)$, is a Brownian motion. Allowing *w*(*t*) to vary in time defines the effective transmission rate $\beta (t)=g^{-1}(w(t))$, which controls the extent to which a disease spreads between individuals within a population.

### State space model

Incorporating temporal information about the dynamics driving the spread of COVID-19 into our model, we discretize the path of *w*(*t*) which defines the transmissibility, and seek to infer *p*(*t*) at each time step. These two correlated time series quantify the rate at which COVID-19 spreads through a population^10,22^. Then coupling these time series with observed case counts as reported by public health agencies, we adopt a state-space modelling paradigm for our inference problem^23^.

A state space model relates two discrete time processes by a probabilistic model incorporating both state evolution and observation densities. In the present context, we are given the number of cases reported by the public health officials, and seek to infer the distribution of the reporting rate *p*(*t*) and of the transmission rate $\beta (t)$. We view the transmission rate and reporting rate as discrete time processes and write $\beta _t$ and $p_t$ for the discrete time counterparts of $\beta (t)$ and *p*(*t*), respectively. Making some regularity assumptions^24^ on the evolution dynamics of the system given in equation (1), and denoting any sequence as $\{c_t\}_{t\ge 0}$ for $i\le j$ as $c_{i:j}= (c_i, c_{i+1},\dots , c_j)$, we view the stochastic epidemiological model as a state space model, written

2 $$\begin{aligned} \begin{aligned} {\mathrm{d}}w_t&= \theta _w\,{\mathrm{d}}B_t, \quad {\mathrm{d}}p_t = p_t {\mathrm{d}}t + \vartheta ^2{\mathrm{d}}W_t,\\ Y_{1:T}&\sim h(Y_{1:T}|X_{0:T}, p_{0:T}, \theta _{Y}), \quad X_{0:T} = {\mathcal {F}}(w_{0:T}; \theta _w). \end{aligned} \end{aligned}$$

The transmissibility $w_t$ of COVID-19 is controlled by the SDE ${\mathrm{d}}w_t = \theta _w\,{\mathrm{d}}B_t$, with Wiener process $B_t$, parameterized by $\theta _w$. The reporting rate $p_t$ evolves according to the SDE ${\mathrm{d}}p_t = p_t{\mathrm{d}}t + \vartheta ^2{\mathrm{d}}W_t$, where $\vartheta$ is the diffusion parameter and $W_t$ is a Wiener process. The reporting rate $p_t$, coupled with the number of observed cases $Y_{t}$, and associated parameters $\theta _{Y_t}$ defines an observation density, $Y_{t} \sim h (\cdot |X_{t}, p_t, \theta _Y)$. We define $X_t$ as the latent number of cases, i.e., $X_t = \int _{t-1}^{t}\,\beta _{\tau }S_{\tau }\frac{I_{\tau }}{N}{\mathrm{d}}\tau$ or $X_t =\int _{t-1}^{t}\, \kappa E_{\tau } {\mathrm{d}}\tau , 1\le t\le T$ for an SIR or SEIR model, respectively, and $X_0\sim \pi _0$ for a prior density $\pi _0$. We write this recursion as ${\mathcal {F}}(w_t;\theta _w)$ to make explicit that the solution to Eq. (1) depends on the state $w_t$ and can be computed for any value thereof via numerical integration.

To define the observation density $h(\cdot |X_t, p_t, \theta _Y)$, we first assume that the reporting of new cases are independent Bernoulli random variables, i.e., each case is reported with probability $p_t$. Then the waiting time until the first reported case is geometrically distributed with the same parameter $p_t$ and we are interested in the weekly incidence rate, conditional on the number of reported cases $Y_t$. Since $Y_t$ is the sum of i.i.d.geometrically distributed random variables with parameter $p_t$, it then follows that $Y_t|X_t\sim {\mathrm {NegBin}}(p_t, X_t)$. Invoking the central limit theorem, the observations $Y_t$ are approximately Gaussian with mean $p_t X_t$ and variance $p_t(1-p_t)X_t + (p_t X_t \eta )^2$. In this scenario, the variance term contains an additional parameter $\eta$, which describes the over-dispersion within a population. See the Appendix for a more detailed discussion about the distribution of $Y_t$. Lastly, we define parameters $\theta _{Y} = (\eta , \vartheta )$ and $\theta _X = (\kappa , \gamma , X_0)$, or $\theta _X = (\gamma , X_0)$, for the SEIR or SIR model, respectively.

### Model parameters

We now describe the parameters utilized in our model. Firstly, the vector $\theta _Y = (\eta , \vartheta )$ contains parameters utilized in our model when investigating a temporally-varying reporting rate $p_t$. Here $\eta$ is incorporated in the observation variance, specifying over-dispersion within the observations, and indicating heterogeneity within a population^25,26^. It is a common occurrence in count data^25,26^ and accounts for large variances in individual outcomes. Furthermore it can signal the presence of ‘super-spreading’ events^27^. Secondly, $\vartheta$ is the standard deviation of the reporting rate. By investigating the marginal density of $p_t$ at each time step, we may quantify the uncertainty in the rate of reporting of positive cases.

We compare our model against a model with constant reporting rate, denoted $p_c$, for the entire time duration. In the model with constant reporting rate, we set $\theta _Y = \eta$, and we may infer the variance *a posteriori* from Markov chain Monte Carlo (MCMC) integration, but this is a static value that is not capable of adapting to changes in the realities of a pandemic, such as more sensitive or accurate testing methods and the availability of individuals to obtain a test.

For the parameters governing the movement between the compartments of the epidemiological model, aside from the transmission rate $\beta _t$, we follow^28^ and assume that the distribution of $\gamma$, governing the movement of individuals from the infected bin to recovered, follows a Gaussian distribution with mean 5.058 days and standard deviation of 1.519 days, with support on the interval of 2.228 and 11.800 days. For our SEIR models, we sample the parameter *k* from a gamma distribution with shape parameter 1.058 and scale 2.174. The parameter *k* controls the rate at which individuals move from the disease’s latent phase to an active infection.

## Results

### Parameter uncertainty

**Figure 1:**
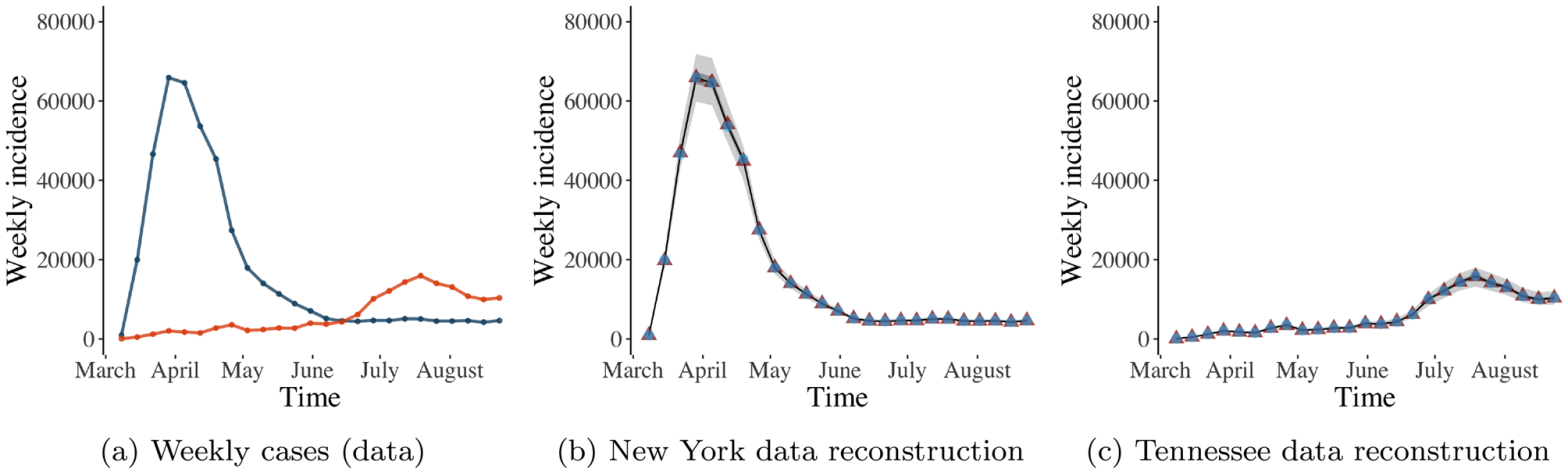
The weekly data of COVID-19 cases for New York (blue) and for Tennessee (orange) from March 1, 2020 through August 31, 2020 are shown in (**a**). The reconstructed weekly cases for New York and for Tennessee over the same time period, based on our SIR model with time-varying reporting rate $p_t$, are displayed in (**b**) and (**c**), respectively. In (**b**) and (**c**), which show our model-based data reconstruction, blue triangles, red triangles, black lines, light-shaded grey areas and dark-shaded grey areas represent the original data, posterior means, posterior medians, $75\%$ credible intervals and $95\%$ credible intervals, respectively.

Two data sets of reported COVID-19 cases, one from New York and one from Tennessee, demonstrate different evolution dynamics, see Fig. 1b,c respectively for a plot of the weekly new case counts. The pandemic went through a period of sustained exponential growth in New York, primarily in the New York City metropolitan region, then abated to a near constant level in subsequent months. Furthermore during the initial wave in New York, testing was not widely available, and the 7-day rolling average of positive test case peaked at nearly 50% in early April 2020^29^. On the contrary, the Tennessee data, which are representative of the evolution dynamics of case counts for the majority of other states, exhibit a slow initial increase, followed by a first wave in April and a much larger increase in July. The variations between the incidence data for these two states are visible in Fig. 1a. These two datasets represent the initial phase of the pandemic from two distinct perspectives. Firstly, the dramatic increase in the NY case counts during March 2020 induces greater uncertainty in the parameters as compared with the gradual increase in the Tennessee case counts. Secondly, the different levels of induced parameter uncertainty enable us to study how such different levels of uncertainty propagate in time through our model.

**Figure 2:**
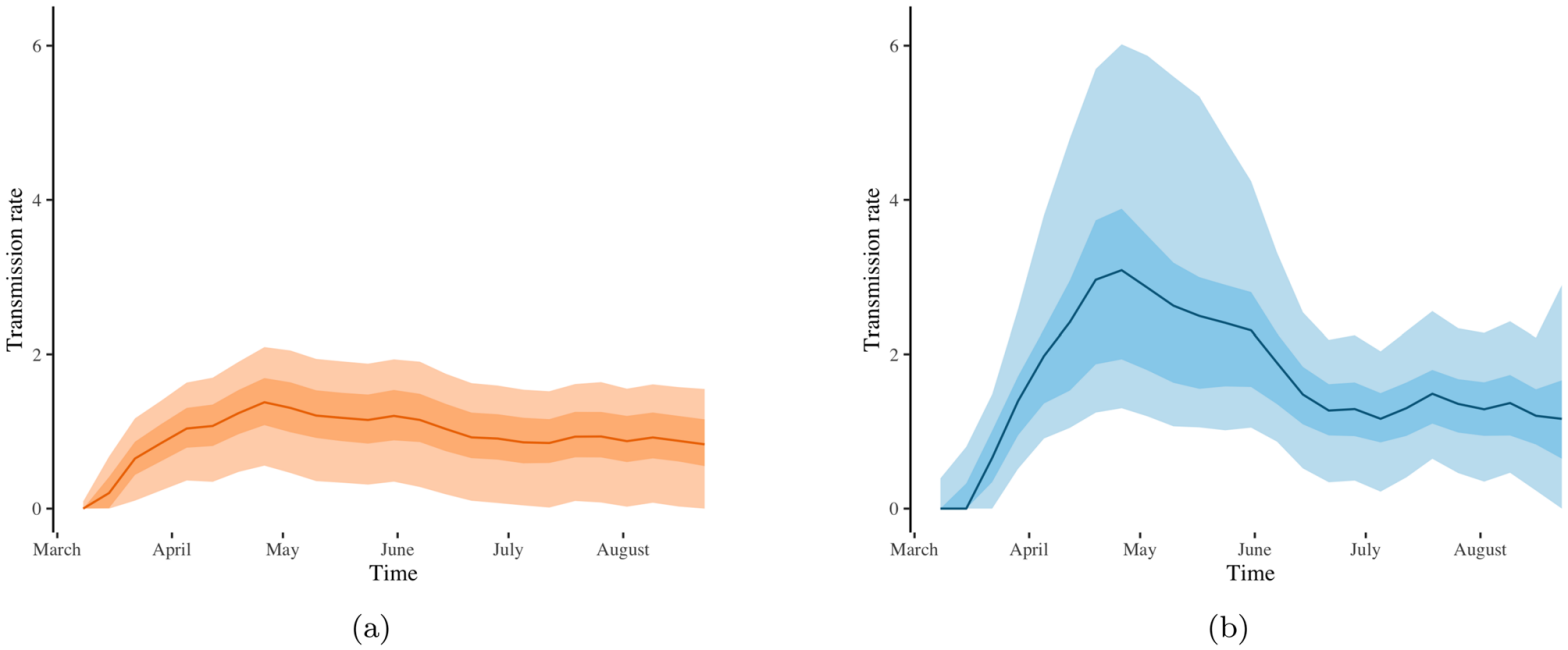
Estimates of the COVID-19 transmission rate for New York based on the typical SIR model with constant reporting rate (**a**) and based on our SIR model with a time-varying reporting rate $p_t$ (**b**). Solid lines, light-shaded and dark-shaded areas correspond to posterior means, $75\%$ and $95\%$ credible intervals of the associated transmission rates.

The estimated transmission rate for New York based on our SIR model with time-varying reporting rate $p_t$ exhibits a spike in transmissibility in April and May (Fig. 2b), which agrees with the spike of observed COVID-19 cases in April and May (blue line in Fig. 1a). The typical SIR model with constant reporting rate produces a transmission rate estimate that fails to capture this spike in transmissibility, as seen in Fig. 2a. Recall that posterior estimates of the transmission rate quantify the rate at which susceptible individuals move from the susceptible compartment to an active stage of infection. As demonstrated by Figs. 1a and 2, our SIR model with time-varying reporting rate provides transmission rate estimates that reflect the observed dynamics of transmission more faithfully than a SIR model with constant reporting rate.

Furthermore, the higher rate of change in the number of observed COVID-19 cases in New York between March and May (blue line in Fig. 1a) induces higher uncertainty in the estimation of the transmission rate. Our SIR model with time-varying reporting rate yields wider credible intervals (Fig. 2b) for the estimated transmission rate in New York between March and May in comparison to the SIR model with constant reporting rate (Fig. 2a). Thus, letting the reporting rate vary with time facilitates the detection of periods of higher uncertainty in transmission rate estimates.

**Figure 3:**
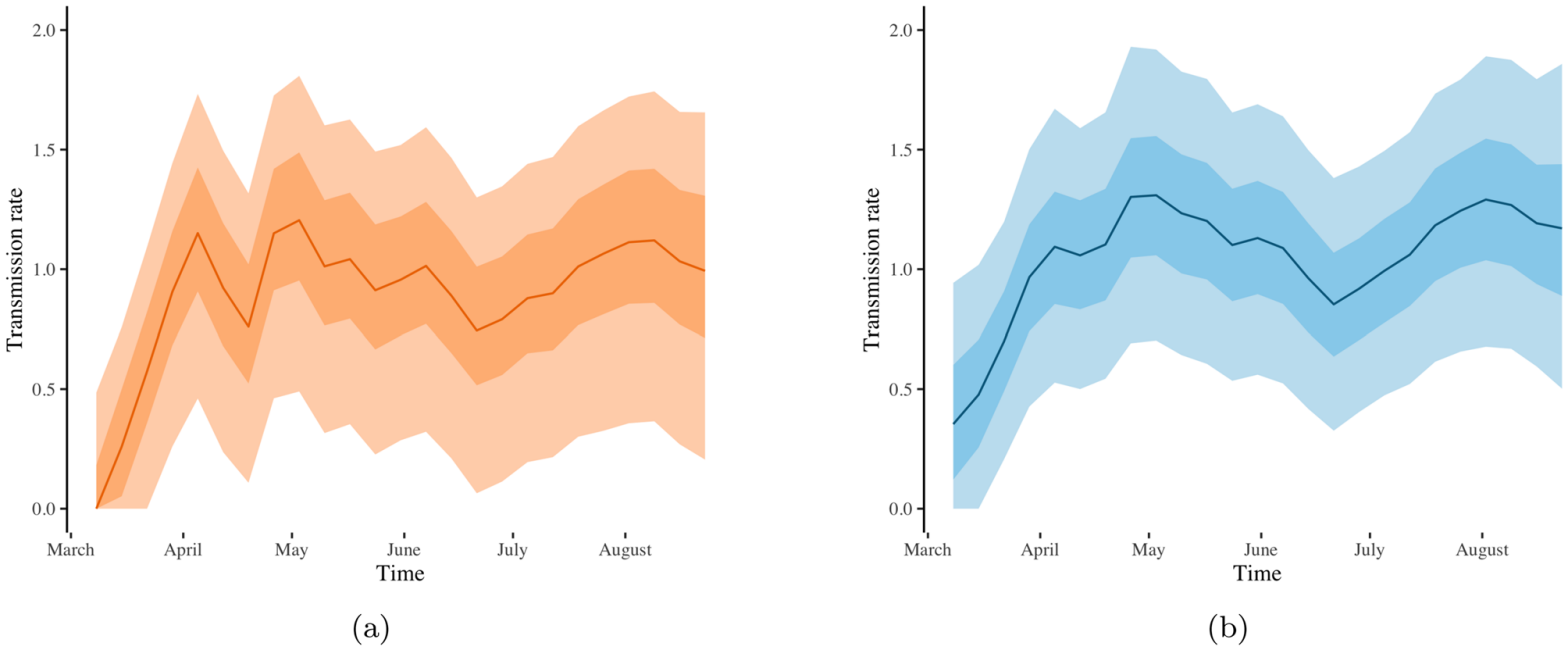
Estimates of the COVID-19 transmission rate for Tennessee based on the typical SIR model with constant reporting rate (**a**) and based on our SIR model with a time-varying reporting rate $p_t$ (**b**). Solid lines, light-shaded and dark-shaded areas correspond to posterior means, $75\%$ and $95\%$ credible intervals of the associated transmission rates.

**Figure 4:**
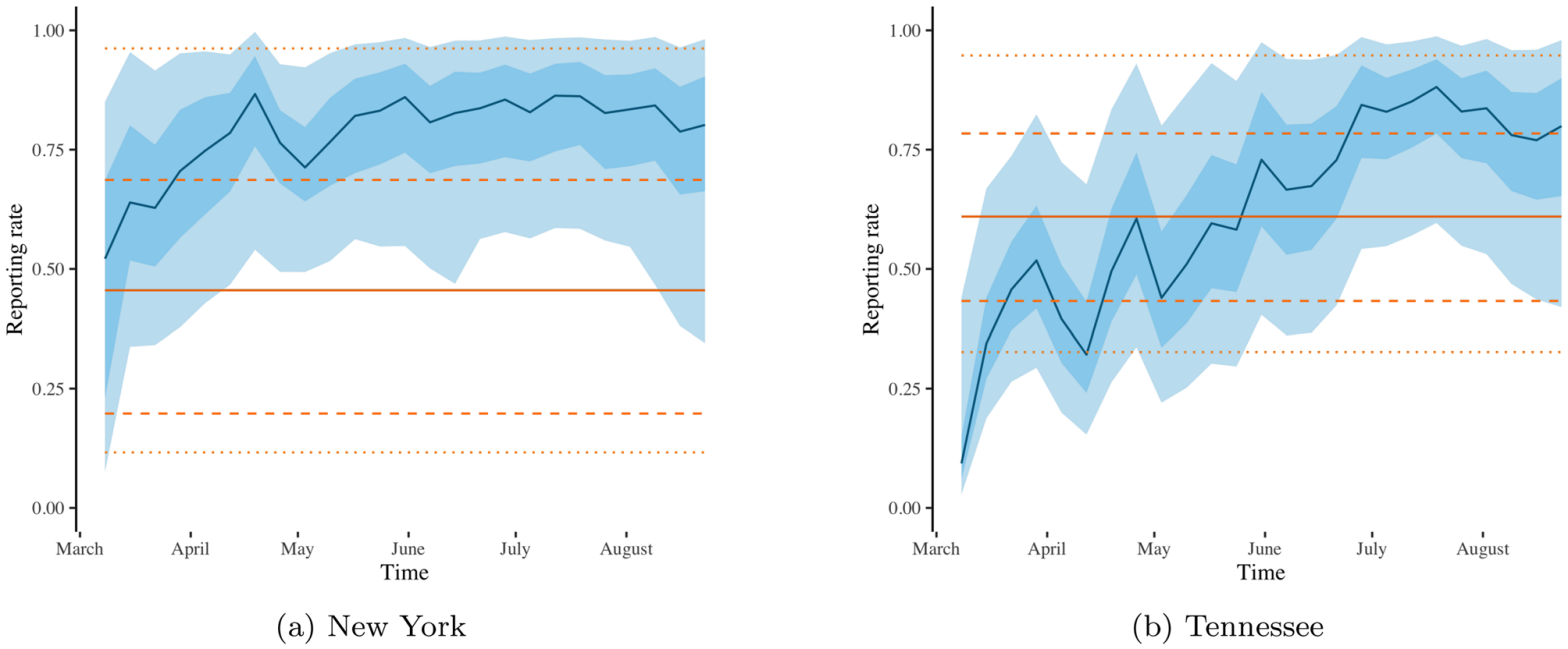
Estimates of the COVID-19 reporting rate for New York (**a**) and for Tennessee (**b**) obtained from the SIR model with constant (orange) and with time-varying (blue) reporting rate. The blue line, light-shaded and dark-shaded blue areas represent the respective posterior mean, $75\%$ and $95\%$ credible intervals of the time-varying reporting rate. The orange solid, orange dashed and orange dotted line represent the respective posterior mean, $75\%$ and $95\%$ credible intervals of the constant reporting rate.

The transmission rate estimates obtained by fitting the SIR model with time-varying and with constant reporting rate to the Tennessee COVID-19 case data agree with one another for the period between May and August (see Fig. 3a,b). However, the SIR model with time-varying reporting rate estimates a smaller drop in the transmission rate in April as compared with the SIR model with constant reporting rate. The former model exhibits a reduced reporting rate estimate in April (blue line in Fig. 4b). So, both models capture the small drop in the number of COVID-19 cases observed in Tennessee during April (orange line in Fig. 1a); the flexible SIR model with time-varying reporting rate attributes this drop to decreased reporting rate (Fig. 4b), whereas the SIR model with constant reporting rate attributes the drop to decreased transmission rate (Fig. 3a). There is no evidence to corroborate which of the two interpretations is correct. Nevertheless, the SIR model with time-varying reporting rate has a wider range of potential options, and it explains the drop in cases via reduced reporting rate in April, which is an explanation not available via the SIR model with constant reporting rate.

Figure 4 presents the estimated reporting rates for the SIR model with time-varying reporting rate (in blue) and with constant reporting rate (in orange). In the case of New York (Fig. 4a), the $75\%$ and $95\%$ credible intervals for the estimated constant reporting rate are wider than the respective credible intervals for the estimated time-varying reporting rate. This indicates that our more flexible SIR model reduces the uncertainty in reporting rate estimation by letting the rate vary with time. The time-varying reporting rate estimates capture an upward trend in reporting rate both in New York (Fig. 4a) and in Tennessee (Fig. 4b), which can be explained by improvements in infrastructure and in available resources to manage the pandemic as time goes by. On the other hand, the SIR model with constant reporting rate can not accommodate such temporal changes in the management and reporting of the pandemic.

As seen in Fig. 4, SIR modelling with constant reporting rate tends to underestimate reporting rates. The disagreement in reporting rate estimation between the SIR models with time-varying and with constant reporting rate is particularly pronounced in the case of New York (Fig. 4b); notice that the straight orange line (constant reporting rate estimate) is lower than the blue line (time-varying reporting rate estimate). The demonstrated underestimation of reporting rates via SIR modelling with constant reporting rate has been previously noted in the literature, and it has been linked to underestimation of the true number of cases and to bias in transmission rate estimation^30^. Our approach based on SIR modelling with time-varying reporting rate provides a principled approach to avoid reporting underestimation, consequently reducing the uncertainty in predictions of number of cases.

### Predictive uncertainty

**Figure 5:**
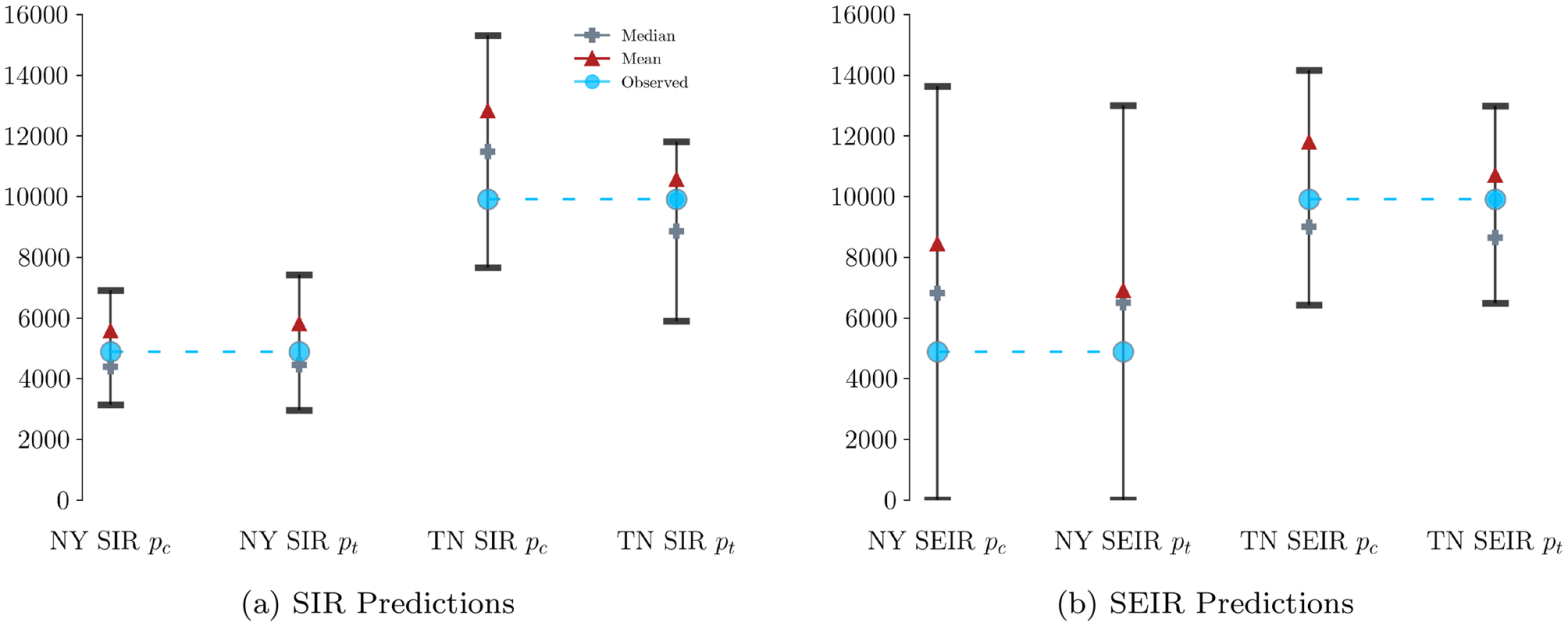
One-week ahead predictions of COVID-19 cases in New York (NY) and in Tennessee (TN) for the week starting August 31, 2020, using SIR and SEIR models with constant reporting rate ($p_c$) or with time-varying reporting rate ($p_t$). Red triangles and grey crosses denote posterior predictive means and posterior predictive medians, respectively. Bars represent $75\%$ posterior predictive intervals. Blue circles depict the observed number of cases.

We make predictions about the number of cases 1 week into the future by fitting SIR and SEIR models with constant or with time-varying reporting rates to New York and to Tennessee data through August 30, 2020. Irrespective of whether constant or time-varying reporting rate is employed, both SIR and SEIR models produce $75\%$ predictive intervals for the number of cases in New York and in Tennessee that contain the observed number of cases (Fig. 5).

Overall, SIR and SEIR models with time-varying reporting rates outperform their counterparts with constant reporting rates in terms of predictive performance (Fig. 5). Firstly, time-varying reporting rates yield narrower predictive intervals, thus reducing predictive uncertainty. Secondly, time-varying reporting rates lead to predictive posterior means that are closer to the observed number of cases. The improved predictive performance attained via modelling based on time-varying reporting rates is observed in three out of the four examined scenarios (SIR and SEIR models fitted to Tennessee data, and SEIR model fitted to New York data), with no apparent improvement in one case (SIR model fitted to New York data).

The wider predictive intervals and therefore higher predictive uncertainty in the Tennessee predictions in comparison to the New York predictions shown in Fig. 5 is seemingly counter-intuitive, given the higher volatility of the New York data (Fig. 1a). However, there is an explanation for the higher predictive uncertainty associated with the Tennessee data. The Tennessee Department of Health changed how they defined an active case on September 3, 2020, resulting in a 1-day decrease of approximately 20, 000 reported cases^31^. Consequently, the predictive distribution is skewed to the left in comparison to the ground truth value, as anticipated after considering the change in the definition of an active case by the Tennessee Department of Health.

## Discussion

The states of New York and Tennessee experienced the first wave of the COVID-19 pandemic in different fashion. Our modelling strategy is able to dynamically adapt to different mitigation strategies enacted in each locality and accurately reflect the course of the pandemic in these geographic regions. We are able to capture the dynamic nature of the transmission rate when intervention methods are enacted, and can quantify changes in the reporting rate of case counts. This modelling strategy yields actionable results for public health officials entrusted with a community’s well-being.

We observe dependence between the time-varying parameters, namely between the transmission and reporting rates, similar to the effects noted by^30^. Indeed, with the significant under-reporting of active cases present in the New York data, a model employing a static reporting rate fails to capture the dynamic nature of COVID-19 transmissibility. As a concrete example, consider the time period from March 1, 2020 through May 24, 2020, when there were 383, 560 active cases reported in New York. Taking the reporting rate inferred by our SIR model, we find that there were 530, 411 active cases, with 95% confidence intervals $(403{,}285,\, 900{,}077)$, a figure which is corroborated by the study of^32^ that identified under-reporting of active COVID-19 cases by considering hospitalization and death rates.

Primarily, a novelty in our modelling approach has been to include a time-varying reporting rate that leads to models which are more likely to fit and explain COVID-19 incidence data. This conclusion is intuitive, since changes in the reporting rate imply changes in the resulting data, so a model with a varying reporting rate is more likely to fit data affected by changes in reporting procedures.

Secondly, we provide a Bayesian approach to quantify uncertainty in relevant epidemiological parameters and in predictions, yielding a source of important information to public health officials tasked with assessing the present state and with suggesting mitigation strategies for subsequent weeks. Our 1-week ahead predictions are accurate, since $75\%$ relevant credible intervals contain the ground truth (Fig. 5a).

The methods described herein are better able to capture not only the time-varying drivers of an epidemic, but also how the reporting of cases changes temporally, thus providing more accurate quantification of the spread of a disease through a susceptible population. Our method provides near real-time actionable information to public health officials, as opposed to methods that use the hospitalization rate^32^ or the excess death rate^33,34^, both of which have a time-lag on the order of weeks. Indeed, previous studies have noted the presence of COVID-19 in February 2020, well before any appreciable increase in hospitalizations^35,36^. Quantifying the spread of a disease through a population and the proportion that are going uncounted by public health agencies is an essential tool for these agencies tasked not only with estimating the proportion of a group that is actively infected, but mitigating the disease’s impact on a population. Indeed, by providing real-time knowledge of the true number of active infections to public health officials, the timing and severity of mitigation strategies can be better informed, thus reducing the community spread of a disease.

While our model cannot capture all the intricacies involved with the public health infrastructure, such as variability of testing sensitivity, access to testing sites, or individuals taking at-home tests that are not reported to public health agencies, we are able to estimate time-sensitive parameters crucial to slowing the spread of an emerging new disease. Indeed, by providing accurate and actionable information about the spread of a disease throughout a population, public health officials could put in place mitigation strategies to slow the spread of a disease.

Future versions of the model could incorporate additional parameters, such as one describing mobility of subpopulations within a geographic region. Such a parameter could capture heterogeneity within a population, and identify those subgroups at higher or lower risk for infection and transmission due to their movements within a specified time window. Lastly, we plan to further investigate the correlation structure between the transmission and reporting rates, to better quantify their dependencies and effects on each other.

## Methods

### Bayesian formulation

Our SIR and SEIR models are parameterized by $\theta = (\theta _w, \theta _X, \theta _Y)$. We factorize the posterior density $\pi (w_{0:T}, p_{0:T},\theta |Y_{1:T})$ of the transmissibility $w_{0:T}$, reporting rate $p_{0:T}$ and model parameters $\theta$, given observations $Y_{1:T}$, as follows:

3 $$\begin{aligned} \pi (w_{0:T}, p_{0:T},\theta |Y_{1:T}) = \pi (w_{0:T},p_{0:T}|Y_{1:T},\theta )\pi (\theta |Y_{1:T}). \end{aligned}$$

According to Eq. (3), we sample from $\pi (w_{0:T}, p_{0:T},\theta |Y_{1:T})$ by alternating between sampling from densities $\pi (w_{0:T},p_{0:T}|Y_{1:T},\theta )$ and $\pi (\theta |Y_{1:T})$ via the particle Markov chain Monte Carlo (PMCMC) algorithm of^37^. PMCMC alleviates issues of convergence and insufficient exploration of the sample space that can arise due to correlations and dependencies between variables.

Sampling from eq. (3) allows us to infer the time-varying transmission rate $\beta _{0:T}$, the time-varying reporting rate $p_{0:T}$, and to make predictions about the future course of the pandemic. Moreover, our Bayesian SIR and SEIR models enable us to quantify the uncertainty of our parameter estimates and of our predictions.

### Particle Markov chain Monte Carlo

To sample from the posterior density of eq. (3), we employ PMCMC sampling^37^. We describe the algorithmic procedure and detail the hyperparameter choices in our model; for an in-depth discussion and theoretical results, see^37,38^. PMCMC alleviates issues with slow MCMC mixing and low acceptance rates that are present in other methodologies for sampling from a joint posterior, such as the pseudo-marginal approach of^39^. First, the sequential Monte Carlo (SMC) procedure is described, followed by PMCMC.

SMC algorithms^38^ provide a way of sampling from distributions defined by state-space models. Based on the decomposition of our posterior density as stated in eq. (3), samples are first drawn from the conditional density $\pi (w_{0:T}, p_{0:T} |Y_{1:T}, \theta )$. The employed SMC algorithm yields a sequence of densities that approximates $\{\pi (w_{0:\tau }, p_{0:\tau }|Y_{1:\tau },\theta ):\tau \ge 0\}$ and the marginal densities $\{{\mathcal {L}}(Y_{1:\tau }|w_{0:\tau }, p_{0:\tau }, \theta ):\tau \ge 0\}$ for a given $\theta$ and $\tau \le T$. SMC first approximates $\pi (w_1, p_1 |Y_1,\theta )$ and ${\mathcal {L}}(Y_1|w_{0:1}, p_{0:1}, \theta )$ by drawing samples from an importance density ${\hat{q}}^{(i)}_1\sim Q_1(\cdot |Y_1, w_1, p_1, \theta )$ for particles $i=1,\dots ,P$ and $q^{(i)}_t := (w^{(i)}_t, p^{(i)}_t)$^37^. SMC approximates $\pi (q_{1:\tau } |Y_{1:\tau },\theta )$ and ${\mathcal {L}}(Y_{1:\tau }|q_{0:\tau },\theta )$, for subsequent iterations $\tau$ by sampling from importance densities ${\hat{q}}^{(i)}_\tau \sim Q_\tau (q_\tau |Y_{1:\tau }, {\hat{q}}^{(i)}_{1:\tau -1}, \theta )$. Requiring these densities to be of the form $Q_\tau (q_{1:\tau }) = Q_{1}(q_{1})\prod _{\tau =2}^T\,Q_\tau (q_\tau |Y_{1:\tau }, q_{1:\tau -1})$, one readily computes an unbiased estimate of the marginal likelihood ${\mathcal {L}}(Y_{1:T}|q_{0:T},\theta )$, which is necessary for the Metropolis-Hastings acceptance ratio in the PMCMC sampler^38^.

Having sampled from $\pi (w_{0:T}, p_{0:T}|Y_{1:T},\theta )$ via SMC, it remains to sample from $\pi (\theta |Y_{1:T})$. At each iteration of the PMCMC algorithm, a value $\theta ^*$ of the parameter $\theta$ is proposed, followed by a sample $\{q_{0:T}^{(i)}\}_{i=1}^P$ generated via SMC. Thus, the problem of sampling from $\pi (w_{1:T}, p_{1:T}, \theta |Y_{1:T})$ is reduced to sampling from $\pi (\theta |Y_{1:T})$, as samples from $\pi (w_{1:T}, p_{1:T} |Y_{1:T}, \theta )$ are obtained via the SMC algorithm.

The model parameters $\eta , w_0, \sigma$, and $p_c$ or $p_t$ are given wide uninformative priors due to the uncertainty about the ongoing pandemic and disparities in reporting data. We model the infection period as a truncated Gaussian distribution with mean of 5.058 days, standard deviation of 1.51, lower bound of 2.228 days and upper bound of 11.8 days, following Lauer et al.^40^. The prior for the latent period $E_t$ is obtained from the study of Moghadas et al.^28^, and is modeled as a gamma distribution with shape and scale parameters 1.058 and 2.174 respectively^41^. For the initial proportions of the population in states $X_0$ we chose a Dirichlet distribution, while constraining the mean of $R_0$ to be ${\mathcal {N}}(0.5, 0.25^2)$, and let the means of the other compartments be equal. By this choice, we ensure that the condition $S_t + E_t + I_t + R_t = N$ or $S_t + I_t + R_t = N$ is satisfied in the respective SEIR or SIR model. Thus, the sum over all compartments in the epidemiological model at each time step is the same as the total population *N*. Lastly, we ran PMCMC sampling with 5, 000 particles and obtain 50, 000 samples from the posterior after a burn-in period of 5, 000 iterations.

### Choice of density for the observational model

A Poisson or a Gaussian approximation can be used for the density $h(Y_{1:T}|X_{0:T}, p_{0:T}, \theta _{Y})$ of the observational model. Pilot PMCMC runs demonstrate similar effective sample sizes for the Poisson and Gaussian approximations, but higher number of particles and therefore higher computational budget are required for the Poisson approximation. For this reason, a Gaussian approximation is preferred.

### Overview of data and of experimental setup

The data used in our experiments are based on daily case counts from March 1, 2020, through August 31, 2020, obtained from the New York Times COVID data repository^13^. In our analysis, we use daily reported case counts and aggregate them on a weekly basis for computational considerations. For one iteration of the PMCMC method, each particle in the ensemble requires the numerical approximation of a system of non-linear ordinary differential equations comprised of *T* time steps. This computational cost becomes infeasible in the case of daily case counts due to the increased number of particles required for PMCMC sampling.

For the implementation of our model and for PMCMC sampling, we use the Bayesian modelling software libBi^42^ and the R packages rbi and rbi.helpers^43,44^. Our models, data and code for reproducing our results can be found at https://github.com/aspannaus/Covid-model.

## Figures and Tables

**Figure 6:**
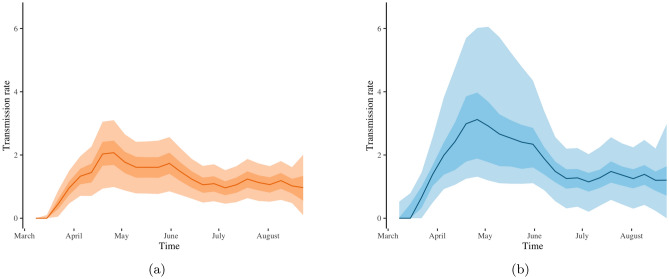
Estimates of the COVID-19 transmission rate for New York based on the typical SEIR model with constant reporting rate (**a**) and based on our SEIR model with a time-varying reporting rate $p_t$ (**b**). Solid lines, light-shaded and dark-shaded areas correspond to posterior means, $75\%$ and $95\%$ credible intervals of the associated transmission rates.

**Figure 7:**
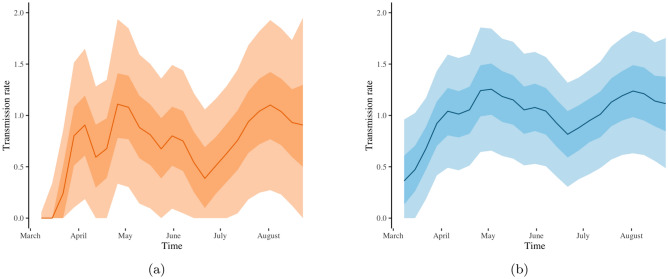
Estimates of the COVID-19 transmission rate for Tennessee based on the typical SEIR model with constant reporting rate (**a**) and based on our SEIR model with a time-varying reporting rate $p_t$ (**b**). Solid lines, light-shaded and dark-shaded areas correspond to posterior means, $75\%$ and $95\%$ credible intervals of the associated transmission rates.

**Figure 8:**
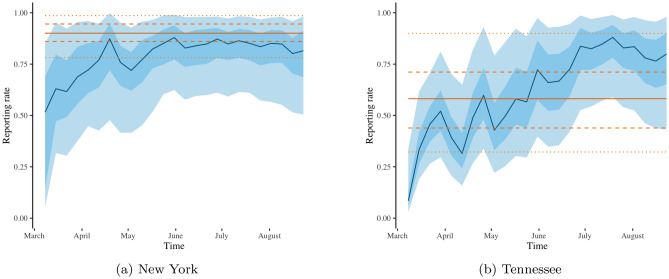
Estimates of the COVID-19 reporting rate for New York (**a**) and for Tennessee (**b**) obtained from the SEIR model with constant (orange) and with time-varying (blue) reporting rate. The blue line, light-shaded and dark-shaded blue areas represent the respective posterior mean, $75\%$ and $95\%$ credible intervals of the time-varying reporting rate. The orange solid, orange dashed and orange dotted line represent the respective posterior mean, $75\%$ and $95\%$ credible intervals of the constant reporting rate.

## Data Availability

The datasets analyzed during the current study are available in the Covid-Model github repository https://github.com/aspannaus/Covid-model.

## SEIR model definition

The SEIR epidemiological model with time-varying transmission rate $\beta (t)$ is represented by the system of differential equations

$$\begin{aligned} \begin{aligned} {\mathrm{d}}S(t)&= -\beta (t)S(t)\frac{I(t)}{N} {\mathrm{d}}t, \\ {\mathrm{d}}E(t)&= (\beta (t)S(t)\frac{I(t)}{N} - \kappa E(t)) {\mathrm{d}}t, \\ {\mathrm{d}}I(t)&= (\kappa E(t) - \gamma I(t)) {\mathrm{d}}t, \\ {\mathrm{d}}R(t)&= \gamma I(t)\, {\mathrm{d}}t,\\ {\mathrm{d}}w(t)&= \mu (w(t), \theta _w){\mathrm{d}}t + \sigma (w(t),\theta _w)\,{\mathrm{d}}B(t), \quad w(t) = g(\beta (t)). \end{aligned} \end{aligned}$$

In this study, we take $\mu =0$ and $\theta _w=\sigma$, thus assuming that the transmission rate on any day is likely to be the same as the previous day, and set $g(\cdot ) = \log (\cdot )$. By choosing $\mu =0$ and $\theta _w=\sigma$, the resulting path *w*(*t*), defined by the stochastic differential equation ${\mathrm{d}}w(t)=\theta _w{\mathrm{d}}B(t)$, is Brownian motion. Allowing *w*(*t*) to vary in time defines the effective contact rate $\beta (t)$, which controls the extent to which a disease spreads between individuals within a population.

## SEIR model results

In this appendix, we present results on parameter uncertainty based on SEIR modelling, while the main manuscript presents results on parameter uncertainty based on SIR modelling. We observe that the inclusion of the latent infected compartment in the epidemiological model has a marked impact on the estimate of the time-varying reporting and transmission rates (Figs. 6, 7 and 8). The effect on the latter follows from recalling that the transmissibility is the product of the probability of passing the disease to another individual and the number of interactions with all individuals. If there is in fact a latent phase in the course of COVID-19, then it follows that individuals in the exposed bin could be mixing with the general population, potentially passing on the disease as it transitions from a latent to active infection.

In the case of New York data, the posterior mean estimate of the constant reporting rate of the SEIR model has narrower credible intervals (straight orange line in Fig. 8a) than the posterior mean estimate of the constant reporting rate of the SIR model (Fig. 4a). However, the credible intervals for the former seem spurious, since the the posterior mean estimate of the time-varying reporting rate of our SEIR model varies substantially with time (blue line in Fig. 8a). In fact, the posterior predictive means of number of cases are closer to the respective observed number of cases, when employing a time-varying (rather than constant) reporting rate in the SEIR model  (Fig. 5b). So, the reduced predictive capacity of the SEIR model with constant reporting rate (in comparison to our SEIR model with time-varying reporting rate) implies further that the estimated constant reporting rate in Fig. 8a is not accurate.

## References

[1] Astolfi R, Lorenzoni L, Oderkirk J (2012). Informing policy makers about future health spending: A comparative analysis of forecasting methods in OECD countries. Health Policy.

[2] Anderson RM, Anderson B, May RM (1992). Infectious Diseases of Humans: Dynamics and Control.

[3] Kermack WO, McKendrick AG (1927). A contribution to the mathematical theory of epidemics. Proc. R. Soc. Lond. Ser. A (Containing papers of a mathematical and physical character).

[4] Blackwood JC, Childs LM (2018). An introduction to compartmental modeling for the budding infectious disease modeler. Lett. Biomath..

[5] Rothe C, Schunk M, Sothmann P, Bretzel G, Froeschl G, Wallrauch C, Zimmer T, Thiel V, Janke C, Guggemos W (2020). Transmission of 2019-nCoV infection from an asymptomatic contact in Germany. N. Engl. J. Med..

[6] Andersson H, Britton T (2012). Stochastic Epidemic Models and Their Statistical Analysis.

[7] Shutt DP, Manore CA, Pankavich S, Porter AT, Del Valle SY (2017). Estimating the reproductive number, total outbreak size, and reporting rates for zika epidemics in South and Central America. Epidemics.

[8] Joh RI, Hoekstra RM, Barzilay EJ, Bowen A, Mintz ED, Weiss H, Weitz JS (2013). Dynamics of shigellosis epidemics: Estimating individual-level transmission and reporting rates from national epidemiologic data sets. Am. J. Epidemiol..

[9] Chong K, Fong H, Zee C (2014). Estimating the incidence reporting rates of new influenza pandemics at an early stage using travel data from the source country. Epidemiol. Infect..

[10] Saberi, M., Hamedmoghadam, H., Madani, K., Dolk, H. M., Morgan, A. S., Morris, J. K., Khoshnood, K. & Khoshnood, B. Accounting for underreporting in mathematical modeling of transmission and control of covid-19 in Iran. *Front. Phys.* **8** (2020).

[11] Sabir Z, Botmart T, Raja MAZ, Sadat R, Ali MR, Alsulami AA, Alghamdi A (2022). Artificial neural network scheme to solve the nonlinear influenza disease model. Biomed. Signal Process. Control.

[12] Ali A, Shah Z, Kumam P (2022). Investigation of a time-fractional covid-19 mathematical model with singular kernel. Adv. Contin. Discrete Models.

[13] Times, N. Y. *New York times covid-19 data* (2020).

[14] Raifman, J., Nocka, K., Jones, D., Bor, J., Lipson, S., Jay, J., Chan, P., Galea, S. *et al.* Covid-19 us state policy database (2020).

[15] Del Moral P, Murray LM (2015). Sequential Monte Carlo with highly informative observations. SIAM/ASA J. Uncertain. Quantif..

[16] Dureau J, Kalogeropoulos K, Baguelin M (2013). Capturing the time-varying drivers of an epidemic using stochastic dynamical systems. Biostatistics.

[17] Funk S, Camacho A, Kucharski AJ, Eggo RM, Edmunds WJ (2018). Real-time forecasting of infectious disease dynamics with a stochastic semi-mechanistic model. Epidemics.

[18] Mishra S, Scott JA, Laydon DJ, Flaxman S, Gandy A, Mellan TA, Unwin HJT, Vollmer M, Coupland H, Ratmann O (2021). Comparing the responses of the UK, Sweden and Denmark to covid-19 using counterfactual modelling. Sci. Rep..

[19] Fisher D, Wilder-Smith A (2020). The global community needs to swiftly ramp up the response to contain covid-19. Lancet.

[20] Lau, H. *et al.* Evaluating the massive underreporting and undertesting of covid-19 cases in multiple global epicenters. *Pulmonology* (2020). 10.1016/j.pulmoe.2020.05.015 PMC7275155 32540223

[21] Oksendal B (2013). Stochastic Differential Equations: An Introduction with Applications.

[22] Gostic KM, McGough L, Baskerville EB, Abbott S, Joshi K, Tedijanto C, Kahn R, Niehus R, Hay JA, De Salazar PM (2020). Practical considerations for measuring the effective reproductive number, Rt. PLoS Comput. Biol..

[23] Birrell PJ, De Angelis D, Presanis AM (2018). Evidence synthesis for stochastic epidemic models. Stat. Sci..

[24] Zhigljavsky A, Zilinskas A (2007). Stochastic Global Optimization.

[25] Bolker BM (2008). Ecological Models and Data in R.

[26] Breslow NE (1984). Extra-Poisson variation in log-linear models. J. R. Stat. Soc. Ser. C (Appl. Stat.).

[27] Lloyd-Smith JO (2007). Maximum likelihood estimation of the negative binomial dispersion parameter for highly overdispersed data, with applications to infectious diseases. PLoS One.

[28] Moghadas SM, Fitzpatrick MC, Sah P, Pandey A, Shoukat A, Singer BH, Galvani AP (2020). The implications of silent transmission for the control of covid-19 outbreaks. Proc. Natl. Acad. Sci..

[29] New York State Department of Health, *Percentage positive results by region dashboard*.

[30] Gamado KM, Streftaris G, Zachary S (2014). Modelling under-reporting in epidemics. J. Math. Biol..

[31] Tennessee Department of Health, *Covid-19 critical indicators*.

[32] Albani V, Loria J, Massad E, Zubelli J (2021). Covid-19 underreporting and its impact on vaccination strategies. BMC Infect. Dis..

[33] Adam D (2022). The pandemic’s true death toll: Millions more than official counts. Nature.

[34] Lau H, Khosrawipour T, Kocbach P, Ichii H, Bania J, Khosrawipour V (2021). Evaluating the massive underreporting and undertesting of covid-19 cases in multiple global epicenters. Pulmonology.

[35] CDC COVID Response Team, Jorden, M. A., Rudman, S. L., Villarino, E., Hoferka, S., Patel, M. T., Bemis, K., Simmons, C. R., Jespersen, M. *et al.* Evidence for limited early spread of covid-19 within the United States, January–February 2020. Morbid. Mortal. Wkly. Rep. **69**, 680 (2020). 10.15585/mmwr.mm6922e1 PMC7315848 32497028

[36] Subramanian, R., He, Q. & Pascual, M. Quantifying asymptomatic infection and transmission of covid-19 in New York city using observed cases, serology, and testing capacity. *Proc. Natl. Acad. Sci.* **118** (2021). 10.1073/pnas.2019716118 PMC7936345 33571106

[37] Andrieu C, Doucet A, Holenstein R (2010). Particle Markov chain Monte Carlo methods. J. R. Stat. Soc. Ser. B (Stat. Methodol.).

[38] Del Moral P, Doucet A, Jasra A (2006). Sequential Monte Carlo samplers. J. R. Stat. Soc. Ser. B (Stat. Methodol.).

[39] Andrieu C, Roberts GO (2009). The pseudo-marginal approach for efficient Monte Carlo computations. Ann. Stat..

[40] Lauer, S. A., Grantz, K. H., Bi, Q., Jones, F. K., Zheng, Q., Meredith, H., Azman, A. S., Reich, N. G. & Lessler, J. The incubation period of 2019-ncov from publicly reported confirmed cases: estimation and application. *medRxiv* (2020). 10.7326/M20-0504 PMC7081172 32150748

[41] He X, Lau EH, Wu P, Deng X, Wang J, Hao X, Lau YC, Wong JY, Guan Y, Tan X (2020). Temporal dynamics in viral shedding and transmissibility of covid-19. Nat. Med..

[42] Murray, L. M. Bayesian state-space modelling on high-performance hardware using libbi, arXiv preprint arXiv:1306.3277 (2013).

[43] Funk, S. *Rbi. helpers: Rbi helper functions* (2016).

[44] Jacob, P. E., Lee, A., Murray, L. M., Funk, S. & Abbott, S. *Rbi: R interface to libbi* (2020).

